# From Effectiveness to Embedding: Developing the Preliminary Mobile Vaccination Embedding Framework for Sub-Saharan African Health Systems

**DOI:** 10.3390/vaccines14100886

**Published:** 2026-10-08

**Authors:** Patrice Ngangue, Abibata Barro, Gbètogo Maxime Kiki, Maurice Ye

**Affiliations:** 1 Faculty of Nursing, Université Laval, Quebec City, QC G1V 0A6, Canada; 2 Institut de Formation et de Recherche Interdisciplinaires en Sciences de la Santé et de L’éducation (Ifrisse), 09 BP 311, Ouagadougou 09, Burkina Faso; fleurbarro03@gmail.com; 3 Département d’ergothérapie, Université de Trois-Rivières, Trois-Rivières, QC G9A 5H7, Canada; gbokiz@gmail.com; 4 World Health Organization, BP 362, Antananarivo 101, Madagascar; yem@who.int

**Keywords:** childhood vaccination, mobile health, mHealth, immunization, sub-Saharan Africa, implementation science, institutionalization, systematic review

## Abstract

Background/Objectives: Mobile phone-based vaccination interventions, including SMS reminders, voice calls and tracking applications, have repeatedly shown effectiveness in controlled settings across sub-Saharan Africa. Many nevertheless stop operating once project funding ends and do not become part of the routine health systems they were intended to strengthen. This study aimed to develop a preliminary evidence-informed conceptual framework explaining the multilevel conditions required to embed mobile vaccination interventions within routine immunization systems in sub-Saharan Africa. Methods: A systematic evidence synthesis was undertaken to inform framework development. Six scientific databases (PubMed/MEDLINE, CINAHL, EMBASE, Epistemonikos, Web of Science and Global Health) were searched for studies published between January 2014 and April 2024. Selection followed the PRISMA 2020 statement, methodological quality was appraised with the Mixed Methods Appraisal Tool (MMAT, 2018 version), and implementation conditions were synthesized thematically through an iterative framework-development process. Results: Eighteen of 913 identified records were included. Four interdependent domains formed the preliminary Mobile Vaccination Embedding (MOVE) framework: user readiness (acceptability, trust and digital literacy among caregivers and providers); technological reliability (network coverage, electricity access and device sustainability); organizational integration (staff training, workflow adaptation and health worker adherence); and system governance (policy frameworks, multisectoral partnerships, financing and integration into national Expanded Programme on Immunization structures). Together, these domains describe the conditions shaping progression from adoption to institutionalization. Conclusions: The primary contribution of this study is the preliminary evidence-informed MOVE framework, which shifts attention from whether mobile vaccination interventions are effective to whether health systems possess the capacity to embed and sustain them. The framework requires prospective refinement, empirical testing and validation across diverse health-system contexts.

## 1. Introduction

Childhood vaccination remains one of the most cost-effective public health interventions available for reducing avoidable morbidity and mortality in children [1,2]. In sub-Saharan Africa, however, decades of investment in immunization programs have not brought coverage to the levels set by the World Health Organization (WHO) [3]. Coverage for the third dose of diphtheria–tetanus–pertussis vaccine (DTP3) stood at approximately 70% in the African region in 2021, twenty percentage points below the 90% global target, and national averages conceal wide disparities between and within countries, including along household wealth lines [4].

Mobile technologies have been proposed as a way of strengthening coverage where access to health services is limited [5]. They allow health systems and caregivers to communicate in near real time, which makes it possible to send appointment reminders, trace defaulters and support the supervision of health workers. Randomized trials conducted in low-resource settings report gains in both the completeness and the timeliness of vaccination. In Ethiopia, infants whose caregivers received text message reminders completed the full schedule more often than those in the control arm [6]; the protocol for that trial had been published by the same team [6]. Comparable effects have been described in Kenya [7,8,9] and Burkina Faso [10], and a meta-analysis of trials conducted in low- and middle-income countries reports consistent gains in uptake, completeness and timeliness [11]. A protocol addressing the same question in Tanzania has also been published [12].

The systematic reviews published so far have mostly assembled barriers and facilitators of mobile vaccination interventions as discrete lists: technical constraints on one side, acceptability issues and enabling conditions on the other [13,14,15,16,17,18]. That approach generated useful early evidence, but it leaves a prior question unanswered: why do some mobile vaccination interventions become embedded in routine immunization systems while others remain pilot projects or stop operating once external funding ends?

The difference between adoption and institutionalization matters for implementation science and for practice alike. An intervention adopted within a controlled trial may produce significant outcomes under study conditions and remain structurally disconnected from the health system that hosted it. Institutionalization, understood here as the process by which an intervention becomes a stable and self-sustaining component of routine service delivery, depends on conditions that reach well beyond initial user acceptance or technical feasibility. It requires alignment across several system levels at the same time.

Existing implementation science frameworks such as the Consolidated Framework for Implementation Research (CFIR) [19] and the Reach, Effectiveness, Adoption, Implementation, and Maintenance (RE-AIM) framework [20] provide broad constructs for understanding implementation across settings, but they were not developed specifically to explain the embedding of mobile vaccination interventions within routine immunization systems in sub-Saharan Africa. MOVE was therefore developed as a context- and intervention-specific synthesis that complements, rather than replaces, established implementation science frameworks by organizing the empirical conditions most directly linked to sustained integration and institutionalization.

This study therefore aimed to develop a preliminary evidence-informed conceptual framework explaining the conditions under which mobile vaccination interventions can become embedded within routine immunization systems in sub-Saharan Africa. To inform framework development, we systematically identified and thematically synthesized empirical evidence on the user, technological, organizational and governance conditions associated with sustained implementation. The guiding question was: what multilevel implementation conditions must be aligned for mobile vaccination interventions to progress from short-term adoption to institutionalization within routine immunization systems?

In this article, MOVE refers exclusively to the Mobile Vaccination Embedding framework and should not be confused with the European Union’s MOVE project on methods for improving vulnerability assessment.

The eligibility criteria were structured using the PICO framework:

- Population (P): children of vaccination age and their caregivers, together with healthcare providers, in sub-Saharan Africa;
- Intervention (I): use of mobile phones to improve vaccination coverage, including SMS reminders, voice call reminders, tracking applications and information campaigns;
- Comparison (C): absence of intervention, or conventional interventions without a mobile technology component;
- Outcome (O): conditions, at user, technological, organizational and governance levels, that determine uptake, sustained implementation and embedding in routine vaccination systems.

## 2. Materials and Methods

The study was designed as a framework-development project informed by a systematic evidence synthesis. The evidence-identification and selection stages followed the PRISMA 2020 statement [21] to ensure methodological rigor and transparency. A comprehensive search strategy was developed and applied to six scientific databases: PubMed/MEDLINE, CINAHL, EMBASE, Epistemonikos, Web of Science, and Global Health. The review was registered in PROSPERO in 2024 (CRD42024528146) under the title “Barriers and facilitators in using cell phones for improving coverage of childhood immunization in sub-Saharan Africa: a systematic review.” The completed PRISMA 2020 checklist is provided as Supplementary File S1.

### 2.1. Definition of Concepts

Vaccine. A biological preparation designed to stimulate the immune system to generate antigen-specific immunity against a pathogen and prevent the disease it causes [3].

Vaccination coverage. The proportion of individuals in a target population who have received a specified vaccine or vaccine series within a defined period [3]. Vaccination coverage is a key indicator used to monitor the performance of immunization programmes and population protection.

Barrier. In this study, a barrier was operationally defined as any individual, technological, organizational or system-level condition that hinders the adoption, sustained implementation or institutionalization of a mobile vaccination intervention. This operational definition was informed by implementation science approaches that conceptualize implementation determinants as multilevel influences on intervention delivery [19].

Facilitating factor. In this study, a facilitating factor was operationally defined as any condition that supports the adoption, continued delivery or integration of a mobile vaccination intervention into routine services. For the purposes of framework development, facilitating factors were interpreted as potential embedding conditions rather than merely determinants of initial uptake.

Institutionalization (embedding). Institutionalization was defined as the process through which a mobile vaccination intervention becomes integrated into routine structures, organizational practices, financing arrangements and governance mechanisms such that its continued delivery no longer depends primarily on temporary project funding or external technical support [22,23,24]. In this manuscript, the terms institutionalization and embedding are used interchangeably.

### 2.2. Inclusion and Exclusion Criteria

Selection was limited to empirical articles published in French or English that examined mobile phone-based interventions for childhood vaccination and reported implementation barriers, facilitating factors, or conditions relevant to their adoption, sustained implementation, or integration within health systems in sub-Saharan Africa.

### 2.3. Study Selection

Records were imported into Rayyan (https://www.rayyan.ai) for screening. Selection proceeded stepwise based on the research question and the eligibility criteria. Titles and abstracts were first screened independently by three reviewers. Records passing this stage were read in full. Disagreements over eligibility were settled by consensus among the reviewers. Eighteen studies were retained (Figure 1): fifteen quantitative and three qualitative.

### 2.4. Data Extraction and Analysis

Rayyan was also used to organize the corpus, remove duplicates and manage the selection process. Data extraction followed a structured grid covering study design, country, population, type of mobile intervention, implementation context, reported conditions (facilitators and barriers) and outcomes.

Thematic synthesis was used to identify patterns across studies. Implementation-related findings were first coded inductively without imposing predetermined categories. Conceptually similar findings were then grouped and compared across studies. The emerging categories were subsequently organized according to the level of the health system at which they operated and examined in relation to the distinction between initial adoption, sustained implementation and institutionalization.

### 2.5. Framework Development

Framework development proceeded iteratively. First, implementation-related findings extracted from each study were examined for recurrent concepts and mechanisms. Second, conceptually similar findings were consolidated into candidate domains. Third, the candidate domains were compared across studies and organized according to the health-system level at which they operated. Fourth, the review team examined how the candidate domains related to the transition from initial adoption to sustained implementation and institutionalization, asking whether each domain represented a distinct condition for embedding and how deficits in one domain could constrain progress despite strengths in others. This process generated four interdependent domains: user readiness, technological reliability, organizational integration, and system governance. These domains are designated as levels (L1–L4) in the tables and figures to indicate the health-system level at which each primarily operates; the numbering does not imply a hierarchy of importance or a mandatory temporal sequence. The preliminary framework was refined through repeated comparison with the included studies and discussion among the review team. It should be understood as an evidence-informed conceptual model requiring prospective testing rather than as a validated implementation theory.

### 2.6. Study Quality Assessment

Methodological quality was appraised with the 2018 version of the Mixed Methods Appraisal Tool (MMAT) [25], chosen because it accommodates qualitative, quantitative and mixed methods designs within a single review. Two reviewers appraised each study independently and resolved discrepancies by discussion. The MMAT yields a profile of methodological strengths and limitations rather than a numerical score. No study was excluded on quality grounds; quality information was instead used to calibrate confidence in individual findings.

## 3. Results

### 3.1. Study Selection

The database searches returned 913 records. After removal of duplicates, 814 titles and abstracts were screened, and 50 articles were read in full. Eighteen met all inclusion criteria and were retained for analysis (Figure 1).

### 3.2. Characteristics of the Included Studies

Ten of the eighteen studies were conducted in Nigeria [26,27,28,29,30,31,32,33,34,35] and four in Ethiopia [6,36,37,38]; Burkina Faso [10], Côte d’Ivoire [24], Zambia [39] and Ghana [40] contributed one each. Six were randomized controlled trials [6,24,27,31,33,40], four cross-sectional surveys [26,28,34,37], three observational studies [30,32,39], three qualitative studies [35,36,38] and two quasi-experimental evaluations [10,29]. SMS reminders were the dominant modality, either alone or combined with voice calls, financial incentives or supplementation services; the remaining interventions involved electronic immunization registries, routine immunization data management tools and group messaging platforms. Table 1 summarizes the characteristics of each study and the MOVE levels it informs.

### 3.3. Methodological Quality

Ten studies were rated good and eight satisfactory (Table 2). Reporting was consistently strong for the clarity of the research question and the adequacy of the data collected. The recurrent weakness across the cross-sectional and observational studies concerned the representativeness of samples and the handling of incomplete outcome data. No study was excluded on quality grounds.

The synthesis generated four conceptually distinct but interdependent domains, which were integrated into the preliminary MOVE framework. The findings are therefore presented by framework domain rather than solely by intervention type or study design. Because implementation outcomes, follow-up periods, and study designs varied substantially across the included studies, the synthesis did not test whether interventions addressing a greater number of MOVE domains achieved better implementation outcomes; this remains an empirical question for future prospective research.

### 3.4. Level 1: User Readiness

User readiness covers the individual-level conditions, among caregivers, parents and frontline health workers, that determine whether a mobile vaccination intervention is accepted, trusted and used consistently over time. Where it is lacking, the demand-side uptake required for institutionalization does not materialize.

#### 3.4.1. Acceptability Among Caregivers

Five studies examined the acceptability of SMS or voice call reminders for childhood vaccination [10,27,29,33,36]. Acceptability was generally high in Nigeria, Ethiopia and Burkina Faso: most caregivers described reminder messages as useful, understandable and compatible with their existing practices. In Ondo State, Nigeria, caregivers attending immunization clinics widely reported being willing to receive SMS reminders for vaccination appointments [26], and in a multi-state survey of rural mothers 90.5% expressed the same willingness [28]. In Ethiopia, acceptability was closely associated with prior exposure to mobile phone-based health communication [36].

Acceptability was not evenly distributed. Differences appeared in education, age and previous experience with mobile phones. Women with limited literacy and older caregivers were more hesitant, less because they objected to the principle than because engaging with text-based content was difficult for them. The distinction between conceptual acceptability (agreeing that a service is useful) and operational readiness (being able to use it in practice) is therefore worth maintaining.

#### 3.4.2. Trust and Perceived Value

Acceptability was bound up with trust, both in the technology and in the health system delivering it. In Côte d’Ivoire and Burkina Faso, caregivers who saw the messages as coming from a recognized health authority acted on them more often [10,24]. Concerns about data privacy and unsolicited messaging reduced engagement in some settings, particularly in urban areas where participants were more exposed to commercial spam [41].

#### 3.4.3. Digital and Health Literacy

Literacy, digital as well as health-related, was identified in several studies as a decisive condition of user readiness. Illiteracy made text-based SMS interventions inaccessible to a substantial share of the target population in rural areas of Ethiopia [6,35,36]. Interventions that adapted messages to local languages, used voice formats or simplified visual content reached consistently wider audiences. Delivery in the caregiver’s mother tongue was among the most frequently reported enabling conditions across contexts, and caregivers with low literacy expressed a clear preference for audio formats [37].

Provider readiness mattered just as much. Whether health workers were comfortable with mobile tools, confident in explaining them to caregivers and motivated to use digital tracking systems shaped how far interventions realized their potential. Studies from Ethiopia and Nigeria found frontline workers supportive of reminder systems when these were seen as reducing administrative burden, and resistant when they were seen as adding tasks without corresponding support [35,36,38].

### 3.5. Level 2: Technological Reliability

Technological reliability covers the technical and infrastructural conditions that determine whether a mobile intervention functions consistently enough to be relied upon within routine service delivery.

#### 3.5.1. Network Coverage and Connectivity

Four studies reported that limited mobile network coverage was a fundamental constraint on implementation [6,10,29,36]. In rural and peri-urban areas of Ethiopia, Nigeria and Burkina Faso, irregular signal meant that messages were often delayed or never delivered, which undermined caregivers’ confidence in the system. The consequence was most serious for time-sensitive reminders, where a failed delivery could translate directly into a missed appointment. Repeated unreliability also eroded health workers’ willingness to maintain the supporting infrastructure.

#### 3.5.2. Electricity Access and Device Sustainability

Access to reliable electricity for charging emerged as a structural constraint, particularly in rural areas [36]. Caregivers with limited access to power described managing battery life around reminder schedules as an additional burden. Some rural facilities lacked the steady supply needed to charge the device banks used in health worker-managed systems. Interventions that included solar charging or worked with community charging points reached further in off-grid settings, although these arrangements added a continuing logistical requirement.

#### 3.5.3. Phone Ownership and Access

Phone ownership has grown across sub-Saharan Africa but remains unevenly distributed. Studies in rural Ethiopia and Kenya found that an appreciable proportion of caregivers owned no mobile phone, shared a handset with other household members, or owned only a basic handset incompatible with smartphone applications [37,42]. Sharing raised practical questions, such as which number to register, and social ones, particularly for women in households where access to the phone was controlled by male relatives. Interventions that presupposed individual ownership reached fewer people than those drawing on community phone pools or on the handsets already used by community health workers.

#### 3.5.4. Maintenance of Technical Systems

Beyond the deployment phase, the functioning of mobile vaccination systems depended on continuing maintenance: software updates, SIM card management, database upkeep and troubleshooting. Several studies observed that technical failures accumulated once interventions moved past the active project period and no mechanism for sustained technical support remained. In Nigeria and Zambia, the quality of health information system data, on which accurate targeting of reminders depends, deteriorated where data entry rested on health workers already carrying heavy workloads [30,39].

### 3.6. Level 3: Organizational Integration

Organizational integration covers the conditions within facilities and health systems that determine whether mobile vaccination interventions are absorbed into existing workflows, staffing structures and service delivery routines. Even where user readiness and technological reliability are adequate, interventions fail to embed when organizations lack the structures to carry them.

#### 3.6.1. Staff Training and Capacity

The capacity of health workers to operate and troubleshoot mobile tools was among the most frequently cited organizational conditions. In Nigeria, Côte d’Ivoire and Ethiopia, the absence of training on mobile health platforms was reported as a leading institutional barrier [24,35]. Workers who received structured training covering platform use, explanation to caregivers and resolution of common problems were considerably more likely to sustain use. Interventions deployed without accompanying training were often discontinued soon after an initial period of enthusiasm, once staff met difficulties they had no means of resolving.

The quality of training mattered as much as its quantity. One-off training events produced competence that decayed quickly, especially where staff turnover was high. Training delivered within continuous supervision cycles, with peer coaching, monthly review meetings and refresher sessions linked to performance data, maintained competence better over time [35,36].

#### 3.6.2. Workflow Integration and Task Burden

Health worker adherence depended largely on whether the intervention was experienced as an addition to an already heavy workload or as a tool that simplified routine tasks. In Ethiopia and Nigeria, workers sustained use of reminder systems when these reduced manual tracking, for instance by automating the identification of defaulters that had previously required lengthy register reviews [6]. Systems requiring double data entry, adding administrative steps, or failing to connect with existing paper registers generated friction that wore adherence down.

#### 3.6.3. Supervision and Management Commitment

Active engagement by managers was repeatedly identified as an enabling condition. Facility managers who promoted the tools, attended training, reviewed usage data in team meetings and helped resolve problems created environments in which staff engagement lasted longer [24,35]. Supervisory arrangements that gave explicit attention to intervention performance, for example regular data review meetings where reminder delivery rates were discussed alongside coverage results, supported organizational learning [30].

#### 3.6.4. Operational Multisectoral Collaboration

Collaboration at facility and district level, and particularly between health authorities and telecommunications operators, was emphasized in two studies [26,36]. Technical reliability improved where telecom partners took part in the early design of message delivery infrastructure. Adoption was faster and more durable where district health managers were partners in intervention governance rather than recipients of it.

### 3.7. Level 4: System Governance

System governance covers the macro-level conditions of policy frameworks, financing mechanisms, integration into national programs and cross-sectoral partnerships. Without them, successes achieved at user, technical and organizational levels remain fragile and geographically confined.

#### 3.7.1. Policy Frameworks and National Integration

The most durable interventions were those written into national Expanded Programme on Immunization (EPI) frameworks, with explicit policy recognition and standard operating procedures. Studies from Nigeria and Ethiopia identified the absence of national guidelines for mobile vaccination as a barrier to institutionalization: without policy anchoring, interventions depended on project-specific protocols that varied between facilities and districts and could be neither scaled nor monitored systematically [26,35].

Interventions formally incorporated into national EPI strategies, with defined roles, responsibilities and performance indicators, proved more resilient to staff turnover, leadership change and funding transitions. Policy integration also allowed alignment with national health information systems, so that reminder data contributed to routine program monitoring instead of remaining in project databases.

#### 3.7.2. Financing and External Dependency

A recurring finding was the vulnerability of these interventions to the end of project funding. Programs financed by international organizations, research grants or time-limited donor schemes frequently ceased at closure, including several that had demonstrated effectiveness. Demonstrated effectiveness is therefore not sufficient for institutionalization, and financial sustainability must be planned from the outset. Studies pointed to three ways of reducing dependency on external financing: incorporation of intervention costs into routine national health budgets, agreements with telecommunications companies to obtain subsidized messaging, and inclusion in national digital health financing frameworks [31,40]. Cost analyses of national eHealth programmes provide the evidence needed for such budget negotiations [43].

#### 3.7.3. Strategic Multisectoral Partnerships

National partnerships between health ministries and telecommunications operators were identified as a governance condition. They made possible preferential messaging rates, technical support agreements and coordinated planning that could not be negotiated at facility or district level. Early engagement of telecom partners in intervention design, so that delivery infrastructure was built for scale rather than retrofitted, and formal memoranda of understanding extending beyond individual project cycles, were both highlighted [26,32].

#### 3.7.4. Data Governance and Health Information Systems

Integration of mobile vaccination data into national health information systems, particularly the District Health Information Software (DHIS2) or its equivalents, emerged as a governance condition with practical consequences. Reminder systems that remained separate from routine health information infrastructure generated parallel data streams, which added to health worker burden and could not feed national program monitoring. In Nigeria and Ethiopia, interventions integrated with DHIS2 showed better long-term data quality and were more readily recognized by national program managers as contributions to routine immunization monitoring [6,35,39].

### 3.8. Synthesis of Implementation Conditions

Table 3 maps the conditions identified at each level onto the studies that document them and states the implication of each level for embedding.

## 4. Discussion

The principal contribution of this study is the development of the preliminary Mobile Vaccination Embedding (MOVE) framework, an evidence-informed conceptual model explaining why some mobile vaccination interventions remain effective pilot projects while others become embedded within routine immunization systems. Rather than treating barriers and facilitators as separate lists, MOVE organizes the evidence into four interdependent domains that together shape institutionalization potential.

MOVE is intended to complement established implementation science frameworks rather than duplicate them. CFIR provides a broad taxonomy of implementation determinants across intervention, inner-setting, outer-setting, individual, and process domains [19], whereas RE-AIM emphasizes reach, effectiveness, adoption, implementation, and maintenance [20]. MOVE has a narrower purpose: it organizes the specific conditions identified in the mobile vaccination literature that appear necessary for interventions to become embedded within routine immunization systems. Its added value is therefore contextual and explanatory, linking user, technological, organizational, and governance conditions directly to institutionalization in this intervention area.

### 4.1. The MOVE Framework

The framework holds that the institutionalization of mobile vaccination interventions in sub-Saharan Africa is governed by conditions operating at four interdependent levels: user readiness, technological reliability, organizational integration, and system governance (Figure 2). The levels are presented in sequence for analytical clarity and to reflect increasingly system-level conditions, but they are neither hierarchical nor ranked by importance. A domain reported more frequently in the literature should therefore not be interpreted as intrinsically more important. Rather, the domains function as mutually reinforcing requirements: a deficit at any one of them may limit embedding potential regardless of what has been achieved at the others.

MOVE is not offered as a validated implementation theory. It should be regarded as a preliminary evidence-informed conceptual framework derived from the available empirical literature. Its current value lies in providing program designers, researchers and health-system managers with a structured way of assessing institutionalization potential before, during and after deployment. Prospective applications are needed to test, refine and validate its domains and their relationships.

### 4.2. User Readiness as a Foundational Condition

The high acceptability reported across studies is necessary but not sufficient. Acceptability indicates whether caregivers and providers will engage with an intervention at the outset; it says nothing about whether that engagement will last or whether the intervention will be absorbed into routine practice. Programs that treat early acceptability rates as a measure of success are therefore liable to overestimate their embedding potential. Work on caregiver preferences points in the same direction: parents in northern Nigeria expressed clear priorities among alternative interventions to improve immunization uptake [44], and qualitative work with caregivers and health workers has shown that the perceived quality of vaccination communication depends on more than the channel through which it travels [45].

Constraints of digital and health literacy carry a design implication: interventions should be built for the least literate member of the target population rather than for the modal user. Voice-based reminders in local languages achieved wider and more equitable reach than text messages in several studies, a finding of direct relevance to settings with high rates of adult illiteracy. Building health literacy alongside technical delivery is a condition of sustained user readiness rather than an optional addition.

### 4.3. Technological Reliability as a Prerequisite for Institutionalization

Connectivity is a prerequisite for institutionalization because it determines whether providers and caregivers can treat the intervention as a predictable part of service delivery. An intervention whose messages arrive intermittently does more than fail to deliver reminders: it damages the trust on which embedding depends. Health workers who meet repeated technical failures maintain fidelity less well, and caregivers who receive unreliable reminders act on them less often. Feasibility work in low-resource settings has made the same point about the practical prerequisites of reminder and recall systems [45].

Assessment of technological infrastructure should therefore precede deployment. Interventions introduced where infrastructure is inadequate are better designed as bridging strategies pending improvement than as durable delivery mechanisms. Where infrastructure is improving quickly, as it is across much of sub-Saharan Africa, staged deployment reassessed against connectivity thresholds is a reasonable compromise.

### 4.4. Organizational Integration as the Point of Transition

The most consistent finding of this synthesis is that organizational integration, and within it staff training, workflow embedding and management commitment, marks the transition between adoption and institutionalization. Interventions rarely fail at the user level, where acceptability is generally high, or at the technical level, where problems can be addressed through investment. They fail at the organizational level, where the absence of sustained support allows initial adoption to decay into abandonment. Experience with digital tracking tools elsewhere is consistent with this: an application for monitoring immunization performance and retrieving defaulters proved operable and well received in a low-income setting, but its value depended on how it fitted the work of the staff using it [46]. Similar observations have been reported in hard-to-reach rural and urban settings outside the region [46], and in the coordination of immunization campaigns, where messaging platforms supported supervision only where reporting lines were already clear [32,47].

The implications for program design follow directly. Training belongs inside ongoing supervision cycles rather than in one-off events. Workflows should be co-designed with frontline staff so that mobile tools reduce rather than add to administrative work. Management commitment must be cultivated through leadership engagement and through performance measurement that makes use of the intervention visible. These organizational investments are not secondary to technical deployment; they determine whether it lasts.

### 4.5. System Governance as the Condition for Scale

Successes at individual, facility and district level remain fragile without governance arrangements that anchor them in national systems. The pattern of effective pilots ceasing at the end of project funding follows directly from the absence of such arrangements. Programs succeed within bounded conditions and fail to institutionalize because no policy, financing mechanism or information system carries them past the project boundary.

Governance conditions should accordingly be assessed and addressed when an intervention intended for scale is designed, not once effectiveness has been shown at small scale. Advocacy for policy recognition, negotiation of sustainable financing, formal partnership agreements with telecommunications operators and deliberate integration with DHIS2 or its equivalents belong to responsible intervention design in this context rather than to an optional enhancement package.

### 4.6. Implications for Practice and Research

For implementers, MOVE provides a pre-deployment checklist. What is the baseline level of user readiness in the target population? How reliable is the delivery infrastructure at present? Which organizational structures are already in place, and which can realistically be built? What governance mechanisms will carry the intervention beyond project funding? Addressing these questions before deployment, and monitoring each level during implementation, would improve the long-term performance of mobile vaccination programs.

For researchers, the clearest evidence gaps concern organizational integration and system governance. User readiness and technological reliability are documented in reasonable depth. Far less is known about which training models, supervision structures and workflow designs sustain health worker engagement over time, or about which policy and financing mechanisms have successfully anchored mobile vaccination within national immunization programs. Validation of MOVE should therefore proceed through prospective application in multiple sub-Saharan African settings, comparative case studies of interventions that did and did not embed successfully, assessment of the content and practical relevance of the domains with implementers and decision-makers, and examination of whether domain-level conditions are associated with maintenance and institutionalization outcomes over time. Longitudinal application alongside established implementation science frameworks would also help determine the framework’s convergent, complementary and explanatory value. Prospective studies with longer follow-up and explicit attention to institutionalization outcomes, rather than to short-term coverage effects, are needed; trials designed in the region offer a starting point [48]. Mobile interventions should also be examined alongside the supply-side and service-delivery strategies with which they interact, such as vaccine stock management [49] and mobile outreach services [50].

Comparative case studies would be particularly valuable. Systematic comparison of interventions that embedded successfully with those that did not, within comparable health systems, would generate the kind of knowledge practitioners need when deciding how to design and scale a program.

### 4.7. Limitations

Several limitations apply. First, the search covered publications from January 2014 onward. The boundary was deliberate. Mobile-cellular subscriptions in Africa exceeded 60 subscriptions per 100 inhabitants by 2013 [51], and mobile health applications across the region were expanding rapidly over the same period [51]; the literature treating mobile vaccination as a program strategy rather than as an isolated feasibility exercise dates largely from that point. The technological context of earlier interventions, built on GPRS networks, unidirectional SMS and feature phones without data connectivity, differs enough from present conditions to make synthesis across the two periods difficult to interpret. Earlier pioneering studies therefore warrant separate treatment. Second, the evidence base was geographically concentrated: 10 of the 18 included studies were conducted in Nigeria and four in Ethiopia, with only one study each from Burkina Faso, Côte d’Ivoire, Zambia and Ghana. This uneven distribution limits transferability of the preliminary framework across the diversity of sub-Saharan African health systems. Third, the small number of studies meeting the criteria, 18 out of 913 records, reflects the emerging state of the evidence and limits the generalizability of the synthesis. Fourth, the predominance of quantitative designs constrains the depth of available information on implementation. In particular, organizational integration and system governance were more often documented through observational and qualitative evidence than through experimental studies. This does not make these domains intrinsically less valid, but it means that their current empirical support is less extensive and requires targeted prospective investigation. Fifth, thematic synthesis involves interpretation, and the four-level structure of MOVE is one defensible reading of the evidence rather than the only one.

Given the limited number and uneven geographical distribution of included studies, MOVE remains a preliminary framework. Its domains and proposed relationships require prospective refinement, empirical testing and validation across diverse health-system contexts. Longitudinal application alongside established implementation science frameworks, such as the Consolidated Framework for Implementation Research (CFIR) [19] or the Reach, Effectiveness, Adoption, Implementation and Maintenance (RE-AIM) framework [20], would provide the evidence needed to assess its explanatory and practical value.

## 5. Conclusions

Mobile phone interventions have clear potential to improve childhood vaccination coverage in sub-Saharan Africa. The evidence synthesized in this study suggests that durable implementation depends on the simultaneous alignment of conditions across four health-system domains. These domains form the preliminary MOVE framework.

The preliminary MOVE framework brings user readiness, technological reliability, organizational integration, and system governance together into a single evidence-informed lens for assessing and strengthening the institutionalization potential of mobile vaccination interventions. Its contribution is to shift the analytical question from what impedes adoption to what enables embedding, while providing a testable structure for future research.

Technology alone, or effectiveness demonstrated in bounded trials, will not be enough. Mobile vaccination interventions are more likely to fulfil their promise when they are designed from the outset for the health systems in which they must operate, with attention to the organizational capacity needed to sustain them and the policy and financing arrangements needed to anchor them. MOVE should therefore be considered a preliminary framework requiring prospective refinement and validation rather than a definitive model.

## Figures and Tables

**Figure 1:**
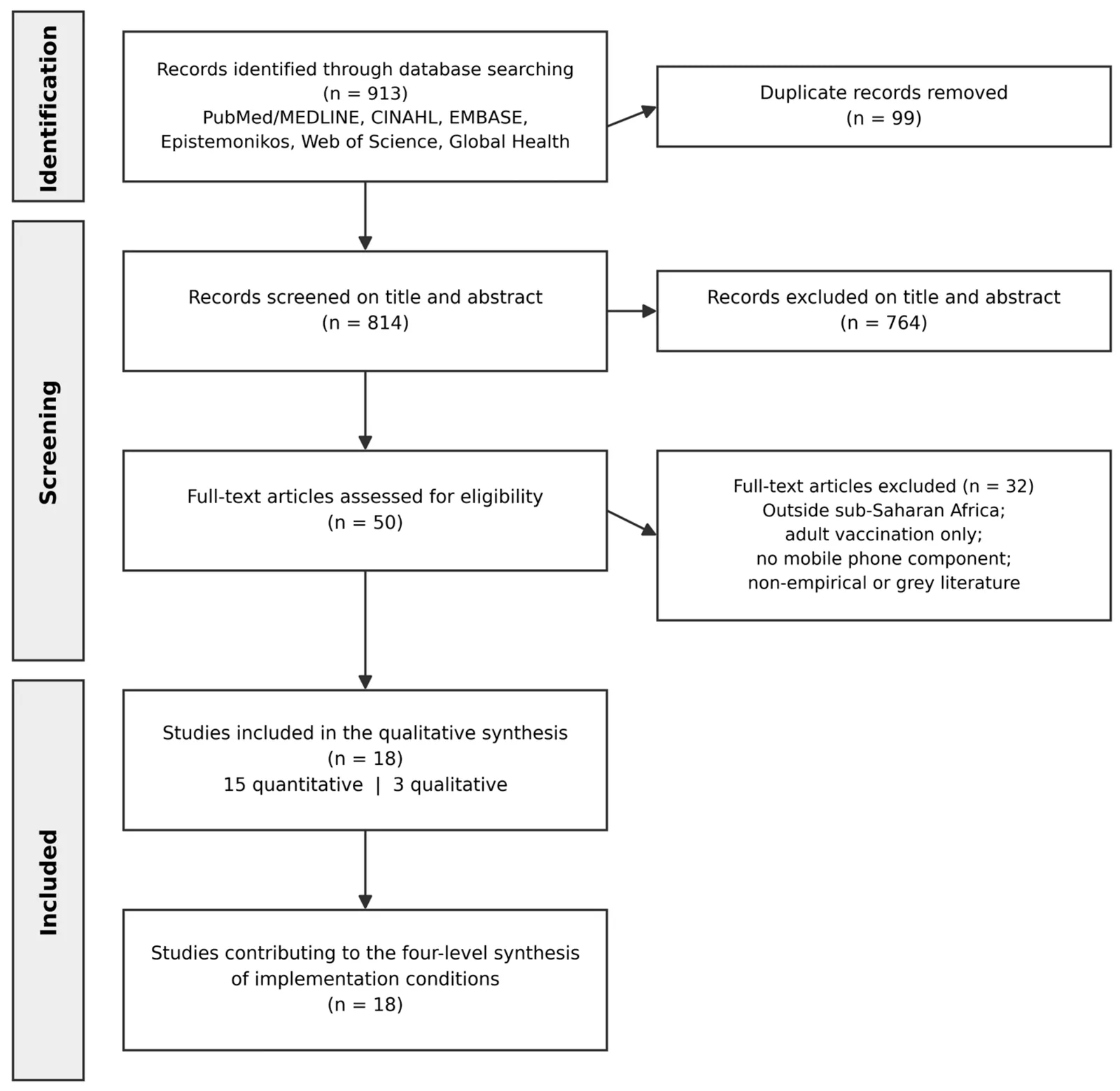
PRISMA 2020 flow diagram of the study selection process.

**Figure 2:**
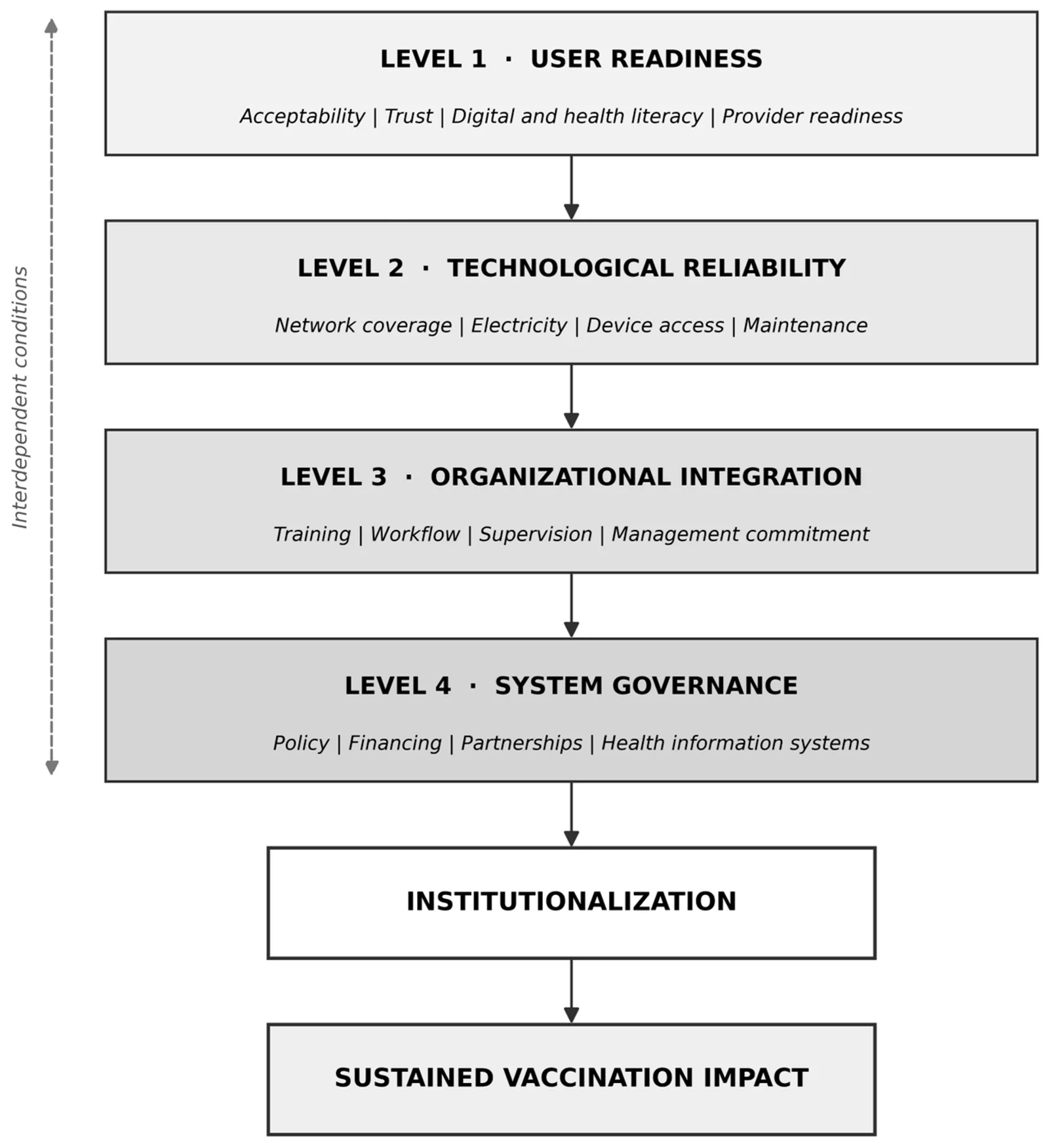
The Mobile Vaccination Embedding (MOVE) framework.

**Table 1:** Characteristics of the 18 included studies. MOVE levels: L1 = user readiness; L2 = technological reliability; L3 = organizational integration; L4 = system governance. RCT = randomized controlled trial; SSA = sub-Saharan Africa; MCH = maternal and child health.

Authors & Year	Country	Study Design	Mobile Intervention	MOVE Level(s)	Key Finding
Akinrinade et al., 2018 [26]	Nigeria	Cross-sectional	Phone ownership and willingness survey	L1	Widespread willingness to receive SMS reminders among caregivers attending immunization clinics
Schlumberger et al., 2015 [10]	Burkina Faso	Quasi-experimental	SMS reminders	L1, L2	Positive effect on vaccination coverage; network reliability a key constraint
Dissieka et al., 2019 [24]	Côte d’Ivoire	RCT	SMS reminders and vitamin A	L1, L3, L4	Higher vaccination coverage; institutional barriers identified at facility level
Ekhaguere et al., 2019 [27]	Nigeria	RCT	Automated calls and SMS	L1, L2	Higher immunization completion; high caregiver acceptability
Oladepo et al., 2019 [28]	Nigeria	Cross-sectional	SMS reminders (knowledge and attitudes)	L1	Knowledge of routine immunization poor but attitudes positive; 90.5% willing to receive reminders
Oladepo et al., 2021 [29]	Nigeria	Quasi-experimental	SMS reminders	L1, L2	Improved immunization completion in rural areas; network reliability a constraint
Clarke et al., 2019 [39]	Zambia	Observational	Electronic immunization registry	L3, L4	Data quality challenges; continuing technical support essential for sustainability
Akerele et al., 2020 [30]	Nigeria	Observational	Routine immunization data management	L3, L4	Staff training and supervision improved data consistency across tools
Kawakatsu et al., 2020 [31]	Nigeria	RCT	SMS appointment reminders	L2, L4	Cost-effective; long-term financing and network infrastructure are prerequisites
Masresha et al., 2020 [32]	Nigeria	Observational	WhatsApp group messaging	L3	Improved coordination; device management and connectivity difficulties noted
Ibraheem R. et al., 2021 [33]	Nigeria	RCT	Call, SMS and SMS-facts reminders	L1, L2	Significant gains in compliance; multi-modal delivery outperformed SMS alone
Ibraheem R.M. et al., 2021 [34]	Nigeria	Cross-sectional	SMS willingness survey	L1	High willingness to receive reminders, shaped by education and phone ownership
Mekonnen et al., 2021a [36]	Ethiopia	Qualitative	SMS reminder system	L1, L2, L3	High acceptability; network, training and institutional support identified as conditions
Mekonnen et al., 2021b [37]	Ethiopia	Cross-sectional	SMS preference and intention	L1	Positive intention; audio formats preferred by caregivers with low literacy
Mekonnen et al., 2021c [6]	Ethiopia	RCT	SMS reminders	L1, L2, L3	Improved completeness and timeliness of vaccination; infrastructure difficulties persist
Levine et al., 2021 [40]	Ghana	RCT	SMS nudges and financial incentives	L1, L4	Improved neonatal vaccination; financing mechanisms and incentive design decisive
Obi-Jeff et al., 2022 [35]	Nigeria	Qualitative	SMS reminder implementation	L3, L4	Training, supervision and policy integration identified as embedding conditions
Gilano et al., 2024 [38]	Ethiopia	Qualitative	mHealth for MCH services	L1, L3	Provider readiness and workflow integration critical for sustained engagement

**Table 2:** Quality assessment using the Mixed Methods Appraisal Tool (MMAT, 2018 version). C1–C5 = applicable MMAT criteria for the relevant study design. ✓ = criterion met; ✗ = criterion not met; ? = insufficient information to assess.

Authors & Year	Study Type	C1	C2	C3	C4	C5	Overall Quality
Akinrinade et al., 2018 [26]	Cross-sectional	✓	✓	?	✓	✓	Satisfactory
Schlumberger et al., 2015 [10]	Quasi-experimental	✓	?	?	✓	✓	Satisfactory
Dissieka et al., 2019 [24]	RCT	✓	✓	✓	✓	✓	Good
Ekhaguere et al., 2019 [27]	RCT	✓	✓	✓	✓	✓	Good
Oladepo et al., 2019 [28]	Cross-sectional	✓	✓	?	✓	✓	Satisfactory
Oladepo et al., 2021 [29]	Quasi-experimental	✓	✓	✓	✓	✓	Good
Clarke et al., 2019 [39]	Observational	✓	✓	?	?	✓	Satisfactory
Akerele et al., 2020 [30]	Observational	✓	✓	?	?	✓	Satisfactory
Kawakatsu et al., 2020 [31]	RCT	✓	✓	✓	✓	✓	Good
Masresha et al., 2020 [32]	Observational	✓	✓	?	✗	✓	Satisfactory
Ibraheem R. et al., 2021 [33]	RCT	✓	✓	?	✓	✓	Good
Ibraheem R.M. et al., 2021 [34]	Cross-sectional	✓	✓	?	✓	✓	Satisfactory
Mekonnen et al., 2021a [36]	Qualitative	✓	✓	✓	✓	✓	Good
Mekonnen et al., 2021b [37]	Cross-sectional	✓	✓	?	✓	✓	Satisfactory
Mekonnen et al., 2021c [6]	RCT	✓	✓	✓	✓	✓	Good
Levine et al., 2021 [40]	RCT	✓	✓	✓	✓	✓	Good
Obi-Jeff et al., 2022 [35]	Qualitative	✓	✓	✓	✓	✓	Good
Gilano et al., 2024 [38]	Qualitative	✓	✓	✓	✓	✓	Good

Overall rating: Good = all five applicable criteria assessed as met; Satisfactory = three or four criteria met. No study was excluded on quality grounds alone.

**Table 3:** Implementation conditions mapped to the Mobile Vaccination Embedding (MOVE) framework. DHIS2 = District Health Information Software 2; EPI = Expanded Programme on Immunization; HIS = health information system.

MOVE Level	Key Conditions	Representative Studies	Embedding Implication
L1—User readiness	Caregiver acceptability; trust in the system; digital and health literacy; provider readiness; language accessibility	[26,29,33,36,37,38]	High acceptability is necessary but not sufficient. Literacy constraints call for audio-based delivery in local languages. Provider readiness must be cultivated rather than assumed.
L2—Technological reliability	Mobile network coverage; electricity access; phone ownership; device sharing; system maintenance	[6,27,29,31,36]	Connectivity is a prerequisite for institutionalization. Infrastructure assessment should precede deployment, and staged rollout is advisable where coverage is low.
L3—Organizational integration	Quality of staff training; continuity of supervision; workflow co-design; management of task burden; management commitment	[6,24,30,32,35,36,38]	The point of transition. One-off training is insufficient; training embedded in supervision cycles and workflow co-design with frontline staff are the main determinants of long-term fidelity.
L4—System governance	National EPI policy integration; sustainable financing; telecom partnerships; DHIS2/HIS integration; data governance	[30,31,35,39,40,43]	Without governance anchoring, local successes remain fragile. Financing sustainability and HIS integration must be addressed at the design stage rather than after effectiveness has been demonstrated.

## Data Availability

All data analysed in this review are contained within the published articles listed in the reference list. The search strategy, screening records and data extraction grid are available from the corresponding author on reasonable request.

## Supplementary Materials

The following supporting information can be downloaded at: https://www.mdpi.com/article/10.3390/vaccines14100886/s1, Supplementary File S1: PRISMA 2020 Checklist [21].

## Abbreviations

The following abbreviations are used in this manuscript:

**Abbreviation**	**Definition**
CFIR	Consolidated Framework for Implementation Research
CINAHL	Cumulative Index to Nursing and Allied Health Literature
DHIS2	District Health Information Software 2
DTP3	Third dose of diphtheria–tetanus–pertussis vaccine
EPI	Expanded Programme on Immunization
HIS	Health information system
MCH	Maternal and child health
MMAT	Mixed Methods Appraisal Tool
MOVE	Mobile Vaccination Embedding
PICO	Population, Intervention, Comparison, Outcome
PRISMA	Preferred Reporting Items for Systematic Reviews and Meta-Analyses
RCT	Randomized controlled trial
RE-AIM	Reach, Effectiveness, Adoption, Implementation, Maintenance
SMS	Short message service
SSA	Sub-Saharan Africa
WHO	World Health Organization
