# Supplementary material for: From Effectiveness to Embedding: Developing the Preliminary Mobile Vaccination Embedding Framework for Sub-Saharan African Health Systems
Source: Vaccines (Basel). 2026 Oct 8;14(10):886. doi: 10.3390/vaccines14100886 (PMC13709480; doi:10.3390/vaccines14100886)
Supplement: Supplementary file 1 [file vaccines-14-00886-s001.zip › vaccines-4534107-supplementary.pdf]

Supplementary File S1: PRISMA 2020 Checklist

From Effectiveness to Embedding: Developing the Preliminary Mobile Vaccination Embedding Framework for Sub-Saharan African Health Systems

Section	Topic	Item #	Checklist item	Location where item is reported
TITLE	Title	1	Identify the report as a systematic review.	p. 1 (article type: "Systematic Review").
ABSTRACT	Abstract	2	See the PRISMA 2020 for Abstracts checklist.	p. 1; see separate PRISMA 2020 for Abstracts checklist.
INTRODUCTION	Rationale	3	Describe the rationale for the review in the context of existing knowledge.	pp. 2–3.
INTRODUCTION	Objectives	4	Provide an explicit statement of the objective(s) or question(s) the review addresses.	p. 3.
METHODS	Eligibility criteria	5	Specify the inclusion and exclusion criteria for the review and how studies were grouped for the syntheses.	pp. 3–4 (PICO and Section 2.2).
METHODS	Information sources	6	Specify all databases, registers, websites, organisations, reference lists and other sources searched or consulted to identify studies. Specify the date when each source was last searched or consulted.	p. 3 (six databases); p. 1 reports the search period as January 2014–April 2024. Exact last-search date for each database is not reported.
METHODS	Search strategy	7	Present the full search strategies for all databases, registers and websites, including any filters and limits used.	Not reported in the main manuscript; the Data Availability Statement (p. 19) states that the search strategy is available from the corresponding author on reasonable request.
METHODS	Selection process	8	Specify the methods used to decide whether a study met the inclusion criteria, including how many reviewers screened each record/report, whether they worked independently, and any automation tools used.	p. 4 (three reviewers independently screened titles/abstracts; full texts assessed; disagreements resolved by consensus; Rayyan used).
METHODS	Data collection process	9	Specify the methods used to collect data from reports, including how many reviewers collected data from each report, whether they worked independently, any processes for obtaining/confirming data from investigators, and any automation tools used.	p. 5 describes the structured extraction grid and variables extracted. Number/independence of data extractors and author-contact procedures are not reported.
METHODS	Data items	10a	List and define all outcomes for which data were sought. Specify whether all compatible results were sought and, if not, the methods used to decide which results to collect.	pp. 3–5 (PICO outcome and implementation conditions; extraction grid).
METHODS	Data items	10b	List and define all other variables for which data were sought and describe assumptions made about missing or unclear information.	p. 5 (study design, country, population, mobile intervention, implementation context, facilitators/barriers and outcomes). Assumptions for missing/unclear information are not reported.
METHODS	Study risk of bias assessment	11	Specify the methods used to assess risk of bias in the included studies, including details of the tool(s) used, how many reviewers assessed each study and whether they worked	p. 6 (MMAT 2018; two reviewers independently; discrepancies resolved by discussion; no study excluded on quality grounds).

Section	Topic	Item #	Checklist item	Location where item is reported
			independently, and any automation tools used.	
METHODS	Effect measures	12	Specify for each outcome the effect measure(s) used in the synthesis or presentation of results.	Not applicable: no meta-analysis or pooled effect estimation was conducted; the review used thematic synthesis of implementation conditions.
METHODS	Synthesis methods	13a	Describe the processes used to decide which studies were eligible for each synthesis.	pp. 4–6 (eligibility criteria, inductive coding, grouping of conceptually similar findings and framework development).
METHODS	Synthesis methods	13b	Describe any methods required to prepare the data for presentation or synthesis, such as handling of missing summary statistics or data conversions.	Not applicable/not reported; no quantitative data transformation or meta-analysis was undertaken.
METHODS	Synthesis methods	13c	Describe any methods used to tabulate or visually display results of individual studies and syntheses.	pp. 5–6; Tables 1–3 and Figures 1–2.
METHODS	Synthesis methods	13d	Describe any methods used to synthesize results and provide a rationale for the choice(s). If meta-analysis was performed, describe the model(s), heterogeneity methods and software used.	pp. 5–6 (inductive thematic synthesis and iterative framework-development process). No meta-analysis was performed.
METHODS	Synthesis methods	13e	Describe any methods used to explore possible causes of heterogeneity among study results.	Not applicable: no statistical synthesis or formal heterogeneity analysis was conducted.
METHODS	Synthesis methods	13f	Describe any sensitivity analyses conducted to assess robustness of the synthesized results.	Not applicable: no sensitivity analysis was conducted.
METHODS	Reporting bias assessment	14	Describe any methods used to assess risk of bias due to missing results in a synthesis.	Not reported / not assessed.
METHODS	Certainty assessment	15	Describe any methods used to assess certainty (or confidence) in the body of evidence for an outcome.	No formal certainty-of-evidence framework was used. p. 6 states that MMAT quality information was used to calibrate confidence in individual findings.
RESULTS	Study selection	16a	Describe the results of the search and selection process, from the number of records identified to the number of studies included, ideally using a flow diagram.	pp. 5–6; Figure 1 (913 records identified; 814 screened; 50 full texts assessed; 18 included).
RESULTS	Study selection	16b	Cite studies that might appear to meet the inclusion criteria, but which were excluded, and explain why they were excluded.	Not reported at the level of individual excluded full-text studies; Figure 1 (p. 5) reports aggregate exclusion reasons.
RESULTS	Study characteristics	17	Cite each included study and present its characteristics.	pp. 6–8; Table 1.
RESULTS	Risk of bias in studies	18	Present assessments of risk of bias for each included study.	pp. 8–9; Table 2 (MMAT 2018).
RESULTS	Results of individual studies	19	For all outcomes, present, for each study: summary statistics for each group (where appropriate) and an effect estimate and its precision, ideally using structured tables or plots.	Table 1 (pp. 6–8) presents study-level key findings relevant to the qualitative/thematic synthesis. Effect estimates and precision were not synthesized because this was not an effect-estimation meta-analysis.
RESULTS	Results of syntheses	20a	For each synthesis, briefly summarise the characteristics and risk of bias among contributing studies.	pp. 8–9 (methodological quality and overview); Sections 3.4–3.8 and Table 3.

Section	Topic	Item #	Checklist item	Location where item is reported
RESULTS	Results of syntheses	20b	Present results of all statistical syntheses conducted. If meta-analysis was done, present summary estimates, precision and heterogeneity.	Not applicable: no statistical synthesis/meta-analysis was conducted.
RESULTS	Results of syntheses	20c	Present results of all investigations of possible causes of heterogeneity among study results.	Not applicable: no formal heterogeneity investigation was conducted.
RESULTS	Results of syntheses	20d	Present results of all sensitivity analyses conducted to assess robustness of synthesized results.	Not applicable: no sensitivity analyses were conducted.
RESULTS	Reporting biases	21	Present assessments of risk of bias due to missing results for each synthesis assessed.	Not assessed / not reported.
RESULTS	Certainty of evidence	22	Present assessments of certainty (or confidence) in the body of evidence for each outcome assessed.	No formal certainty-of-evidence assessment was performed; study-level methodological quality is reported in Table 2 (pp. 8–9).
DISCUSSION	Discussion	23a	Provide a general interpretation of the results in the context of other evidence.	pp. 14–17.
DISCUSSION	Discussion	23b	Discuss any limitations of the evidence included in the review.	pp. 17–18 (Section 4.7, including geographic concentration, small evidence base and predominance of quantitative designs).
DISCUSSION	Discussion	23c	Discuss any limitations of the review processes used.	pp. 17–18 (date restriction and interpretive nature of thematic synthesis).
DISCUSSION	Discussion	23d	Discuss implications of the results for practice, policy, and future research.	p. 17 (Section 4.6) and pp. 17–18.
OTHER INFORMATION	Registration and protocol	24a	Provide registration information for the review, including register name and registration number, or state that the review was not registered.	p. 3: PROSPERO, CRD42024528146 (registered in 2024).
OTHER INFORMATION	Registration and protocol	24b	Indicate where the review protocol can be accessed, or state that a protocol was not prepared.	PROSPERO registration is reported on p. 3 (CRD42024528146); a separate protocol-access location is not stated in the manuscript.
OTHER INFORMATION	Registration and protocol	24c	Describe and explain any amendments to information provided at registration or in the protocol.	Not reported in the manuscript.
OTHER INFORMATION	Support	25	Describe sources of financial or non-financial support for the review, and the role of the funders or sponsors.	p. 19: "This research received no external funding."
OTHER INFORMATION	Competing interests	26	Declare any competing interests of review authors.	p. 19: authors declare no conflicts of interest.
OTHER INFORMATION	Availability of data, code and other materials	27	Report which review materials are publicly available and where they can be found.	p. 19: data analysed are contained in the cited articles; search strategy, screening records and extraction grid are available from the corresponding author on reasonable request.

Reference: Page MJ, McKenzie JE, Bossuyt PM, et al. The PRISMA 2020 statement: an updated guideline for reporting systematic reviews. BMJ. 2021;372:n71.
